# Identification of a novel subpopulation of Caspase-4 positive non-small cell lung Cancer patients

**DOI:** 10.1186/s13046-020-01754-0

**Published:** 2020-11-13

**Authors:** Michela Terlizzi, Chiara Colarusso, Ilaria De Rosa, Pasquale Somma, Carlo Curcio, Rita P. Aquino, Luigi Panico, Rosario Salvi, Federica Zito Marino, Gerardo Botti, Aldo Pinto, Rosalinda Sorrentino

**Affiliations:** 1 Department of Pharmacy (DIFARMA), University of Salerno, Italy and ImmunePharma s.r.l., Via Giovanni Paolo II 132, Fisciano, 84084 Salerno, Italy; 2 ImmunePharma srl, Department of Pharmacy, University of Salerno, 84084 Fisciano, SA Italy; 3 Anatomy and Pathology Unit, Ospedale dei Colli, AORN, “Monaldi”, Naples, Italy; 4 Thoracic Surgery Unit, Ospedale dei Colli, AORN, “Monaldi”, Naples, Italy; 5 Pathology Unit, Department of Mental and Physical Health and Preventive Medicine, University of Campania “Luigi Vanvitelli”, Naples, Italy; 6 Scientific Direction IRCCS National Cancer Institute “G. Pascale”, Naples, Italy

**Keywords:** Lung cancer, Caspase-4, K-Ras, cMyc, Survival rate, Oncoprotein, Cell proliferation, Tumor progression

## Abstract

**Background:**

Therapy/prognosis of Non-Small Cell Lung Cancer (NSCLC) patients are strongly related to gene alteration/s or protein expression. However, more than 50% of NSCLC patients are negative to key drugable biomarkers.

**Methods:**

We used human samples of NSCLC and mouse models of lung adenocarcinoma.

**Results:**

We showed that caspase-4 was highly present in the tumor mass compared to non-cancerous human tissues. Interestingly, the orthologue murine caspase-11 promoted lung carcinogenesis in mice. Carcinogen-exposed caspase-11 knockout mice had lower tumor lesions than wild type mice, due to the relevance of caspase-11 in the structural lung cell as demonstrated by bone marrow transplantation and adoptive transfer experiments. Similarly to what observed in mice, caspase-4 was correlated to the stage of lung cancer in humans in that it induced cell proliferation in a K-Ras, c-MyC and IL-1α dependent manner. Caspase-4 positive adenocarcinoma (79.3%) and squamous carcinoma (88.2%) patients had lower median survival than patients who had lower levels of caspase-4. Moreover, PD-L1 expression and gene mutation (i.e. EGFR) were not correlated to caspase-4 expression. Instead, NSCLC patients who had K-Ras or c-MyC gene alteration were positively correlated to higher levels of caspase-4 and lower survival rate.

**Conclusions:**

We identified a subgroup of NSCLC patients as caspase-4 positive among which double and triple positive caspase-4, K-Ras and/or c-MyC patients which prognosis was poor. Because K-Ras and c-MyC are still undrugable, the identification of caspase-4 as a novel oncoprotein could introduce novelty in the clinical yet unmet needs for NSCLC patients.

**Supplementary Information:**

**Supplementary information** accompanies this paper at 10.1186/s13046-020-01754-0.

## Background

Lung cancer is one of the leading causes of cancer-related death worldwide [1, 2]. Epidemiological studies report that lung chronic inflammation initiate/promote the development of lung cancer, possibly in conjunction with tobacco use and/or other environmental pollutants (i.e. asbestos, silica, diesel exhaust). Epithelial cells, alveolar macrophages (MФ) and resident dendritic cells (DCs) are the first line of defense for the respiratory tract. Their prolonged contact with insulting exogenous molecules can initiate and sustain inflammatory responses which signature could be IL-1β dependent [3, 4], leading to chronic inflammation [3]. In support, elevated serum levels of C-reactive protein (CRP) and high erythrocyte sedimentation rate (ESR) are both associated to lifestyle (i.e. smoking, air pollutant exposure) and are related to increased risk of lung cancer [5]. Concomitantly, high levels of the pro-inflammatory cytokines, such as IL-1β and IL-18, are detected in the plasma and tissue of lung cancer patients [6], identified as bad prognostic biomarkers for cancer patients [7]. IL-1-like cytokines (i.e. IL-1α, IL-1β, IL-18 and IL-33) are identified as ‘alarmins’. Their expression is tightly regulated by multiprotein complexes referred to as ‘inflammasomes’, which activation promotes caspase-1 cleavage into its active form with the ensuing activation of IL-1β and IL-18 [8]. Alternatively, non-canonical inflammasome engages caspase-11 (also known as caspase-4 in humans) which can induce the release of alarmins such as IL-1α, IL-1β, IL-18 and HMGB1 [8]. Human caspase-4, as well as the analogue murine caspase-11, was described as a pro-inflammatory caspase that can serve as host defense via the induction of pyroptosis to eliminate intracellular pathogens, and via the release of pro-inflammatory IL-1-like cytokine (i.e. IL-1α and IL-1β, IL-18) in a canonical inflammasome pathway. Nevertheless, in this latter case it was demonstrated that caspase-11 unlikely processes IL-1β and IL-18 in a direct manner [9], rather, it can promote the downstream caspase-1 activation via NLRP3 [9]. On the other hand, IL-1α release can be directly related to caspase-11 [10].

In our previous murine study, we reported that tumor-associated macrophages (TAMs) populated lung tumor lesions exerting a pro-tumor activity in a caspase-11/caspase-1-dependent manner, implying that the activation of the inflammasome in TAMs was pro-tumorigenic [11]. Moreover, we found that NSCLC patients had higher circulating levels of caspase-4 than healthy subjects [12]. In this study we demonstrated that caspase-4 was highly present in the tumor mass compared to non-cancerous tissues of NSCLC patients and was responsible for cell proliferation, suggesting it as a novel oncoprotein that collaborates with c-MyC and K-Ras to promote lung cancer, affecting patients’ survival rate.

## Materials and methods

### Human samples

Samples in this study were obtained by patients diagnosed of operable NSCLC (stage IA-IB, *n* = 79; Stage IIA-IIB, *n* = 34; Stage IIIA-IV, *n* = 12), and underwent surgical resection at Ospedale dei Colli, AORN, Monaldi, Naples, Italy, during the period 2014–2017. Clinical data were obtained from questionnaires and histology reports from the Pathological Anatomy Unit of the hospital. The project was approved by the institutional review board and by the Ethical Committee (approval number for lung cancer patients 1254/2014). Samples from lung cancer patients were collected after oral and written information provided by the MDs, and after the signature of a consent form before entering the project. Samples were collected and used within 24 h. Lung cancer patients were 60 ± 10 (mean ± S.E.M.) years of age. Biochemical analyses on PD-L1 and genetic mutations (i.e. EGFR mutations, ALK, ROS1 and MET genetic alterations) were performed by double blinded operator at Ospedale dei Colli, AORN, Monaldi, Naples, Italy. Survival data was analysed according to the period 2014–2017 and was related to the values of tissue caspase-4 after surgical resection. Human samples were collected and processed within 24 h surgical resection.

### Mice

Female specific pathogen-free C57BL/6 mice, B6N.129S2-Casp1 < tm1Flv>/J, CASP1/11 knockout (ko), C3H/HeJ (6–8 weeks of age) (Jackson Laboratories, USA, and Charles River Laboratories, Lecco, Italy) were fed a standard chow diet and housed under specific pathogen-free conditions at the University of Salerno, Department of Pharmacy. CASP11 ko were kindly provided by Dr. Vishva Dixit from Genentech USA. Samples from transgenic mice K-Ras^LA1^ or K-Ras^LA1^/p53^R172HΔ^ were kindly provided by Dr. Quaglino, University of Turin, Italy. All animal experiments were performed under protocols that followed the Italian and European Community Council for Animal Care (2010/63/EU). This study was carried out in strict accordance with the recommendations in the Guide for the Care and Use of Laboratory Animals of the National Institutes of Health. The protocol was approved by the Committee on the Ethics of Animal Experiments of the University of Salerno and by National Institutes of Health with the approval number 13786/2014.

## Experimental protocol

### Mouse model of lung carcinogenesis

Mice were intratracheally (i.t.) instilled with N-methyl-N-nitroso-urea (NMU) at the dose of 50 μg/mouse at week 1 (day 0), week 8 (day 56), week 12 (day 84), and the dose of 10 μg/mouse was instilled at week 1 (day 7), week 2 (day 14), week 9 (day 63), week 10 (day 70), week 13 (day 91) week 14 (day 98), according to Fig. 4a. Lungs were isolated and digested with 1 U/mL collagenase (Sigma Aldrich, Milan, Italy). Cell suspensions were passed through 70 μm cell strainers, and red blood cells were lysed. Cell suspensions were used for flow cytometric analyses of different cell subtypes. Broncho-alveolar lavage fluid (BAL) was collected using 0.5 ml of PBS containing 0.5 mM EDTA and cell counts performed. In addition, lungs were homogenized and cytokines measured.

### Bone-marrow (BM) transplantation

Bone-marrow (BM) transplant experiments were performed using wild type (wt, C57Bl/6 mice) and Caspase-11 ko mice. BM-derived cells were isolated from euthanized donor mice. Recipient 6–8 weeks old mice were irradiated with one dose of 10Grad to deplete endogenous BM stem cells and most of the BM-derived cells, before the transplantation of 1 × 10^6^ donor BM cells, injected into the tail vein of recipient irradiated mice.

Animals were divided in four groups:

1. wt into wt: donor wt cells into recipient wt mice;
2. ko into ko: donor Caspase-11 ko cells into recipient Caspase-11 ko mice;
3. wt into ko: donor wt cells into recipient Caspase-11 ko mice;
4. ko into wt: donor Caspase-11 ko cells into recipient wt mice.

The degree of chimerism was assessed by FACS analysis of CD45.1^+^ blood leucocytes 7–8 weeks after BM transplant. NMU or vehicle were instilled starting at 8 weeks post BM transplant and chimera mice were sacrificed 28 days after the first NMU exposure.

### Flow Cytometry analysis

Cell suspensions obtained by collagenase digested lungs were analysed to evaluate the infiltration and the nature of immune cells recruited to the lung of mice. Cell suspensions were labelled with specific antibodies (CD11b, Gr-1, CD4, CD25, FoxP3).

### Western blotting analysis

Lung homogenates were used to examine the expression of caspase-4, in humans, (ImmunePharma srl, Italy) or caspase-11, in mice (Santa Cruz Technologies, CA, USA), kRas (AbCam, Cambridge, UK) by means of SDS- or Native-PAGE. Data were analysed by means of ImageJ (NIH, USA).

### ELISAs

The presence of tissue caspase-4 was detected by an ELISA kit patented by ImmunePharma s.r.l. (RM2014A000080 and PCT/IB2015/051262) (Department of Pharmacy, University of Salerno, Italy). Custom antibodies were projected by ImmunePharma s.r.l., and they are not currently commercially available. The diagnostic performance of the custom antibodies has been previously described [12]. Tissue caspase-4 expression was compared to caspase-5 by means of ELISA. IL-1α and IL-1β were measured in BAL or lung homogenates as specified in the text, using commercially available ELISA kits (eBioscience, CA, USA). In the first case cytokine levels were expressed as pg/ml in BAL samples, whereas in lung homogenates as pg/mg protein.

### Immunohistochemistry

Human samples of lung tumor were embedded in paraffin to perform tissue microarray (TMA) Patient’s characteristics are reported in Table 1. NSCLC patients were considered as Caspase-4 positive (+) (Table 1) according to a histological score that was calculated by a blinded and certified pathologist at the National Cancer Institute “Fondazione G. Pascale” (Naples, Italy). In particular, positive score was defined as positive area to caspase-4 detection that resulted ≥25% compared to negative area (≤25%). Human samples analysed by TMA were different from those for whom survival rate is described. A custom antibody against caspase-4, provided by ImmunePharma srl, Italy, was used to perform immunohistochemistry analyses. The diammino-benzidinic acid (DAB) system was used to detect complexes. Mouse IgG was used as an isotype control (ImmunePharma srl, Italy).

**Table 1:** Characteristics of NSCLC patients and quantification of Caspase-4 positive (+) vs Caspase-4 negative (−) tissues according to the histological score

	N. patients	Caspase-4 +	Caspase-4 -
**Age**			
≥ 60 yrs	67	53 (79.1%)	14 (20.9%)
≤ 60 yrs	22	15 (68.2%)	7 (31.8%)
**Gender**			
Male	55	40 (72.7%)	15 (27.3%)
Female	34	25 (73.5%)	9 (26.4%)
**Stage I**	39	31 (79.5%)	8 (20.5%)
**Stage II**	25	16 (64%)	9 (36%)
**Stage III**	25	22 (88%)	3 (12%)
**Hystotype**			
Adenocarcinoma	54	39 (72.2%)	15 (27.8%)
Squamous	32	23 (71.9%)	9 (28.1%)
Other	3	2 (66.6%)	1 (33.4%)

Mice left lung lobes were fixed in OCT medium (Pella Inc., Milan, Italy) and 7 μm cryosections were cut. H&E staining was performed and used to measure the tumour burden. Tumor lesions were analysed by means of Image J (NIH, USA) and expressed as Tumor lesions = ratio tumor area/total lung area, as already reported [13]. Lung tumor area and the hyperplastic cells were counted by using serial lung cryosections in a blinded fashion.

## Reverse transcriptase-polymerase chain reaction and real-time polymerase chain reaction

Total RNA was isolated from lung tissue samples by using the RNeasy Mini extraction kit according to the manufacturer’s instructions (Qiagen, United Kingdom). Reverse Transcription was performed by using first-strand cDNA synthesis kit (Qiagen, United Kingdom) followed by PCR, as already reported [14]. Thermal cycling conditions for caspase-4 were 5 min at 95 °C, followed by 45 cycles of 45 s at 94 °C, 30 s at 60 °C, 30 s at 72 °C.

Thermal cycling conditions for c-MyC were 5 min at 95 °C, followed by 45 cycles of 45 s at 94 °C, 30 s at 66 °C, 30 s at 72 °C.

Primer pairs were as follow:

-Caspase 4 (NM_001225.3): Forward 5′-TTTCTGCTCTTCAACGCCAC-3′; Reverse 5′-AGTCGTTCTATGGTGGGCAT-3′;

-c-MyC (REF NM_002467): Forward 5′-AAAGGCCCCCAAGGTAGTTA-3′; Reverse 5′-GCACAAGAGTTCCGTAGCTG-3′.

-β-actin: Forward 5′-AGAGCTACGAGCTGCCTGAC-3′; Reverse 5′-AGCACTGTGTTGGCGTACAG-3′.

RT-PCR for k-Ras was performed according to the MGBE probe following manufacturer’s instructions (PrimeTime Gene expression Mastermix kit, IDT, USA) were 3 min at 95 °C, followed by 50 cycles of 30 s at 95 °C, 30 s at 54 °C, 30 s at 80 °C. Primers were as follows:

k-RAS^G12C^: Forward 5′-AATATAAACTTGTGGTAGTTGGAGCCT-3′.

k-RAS^G12D^: Forward 5′-AAACTTGTGGTAGTTGGAGCGGA-3′.

k-RAS^G12V^: Forward 5′-AAACTTGTGGTAGTTGGAGCAGT-3′.

k-RAS: Reverse 5′-CATATTCGTCCACAAAATGATTCTG-3′.

Probe: 5′−/56-FAM/CTGTATCGTCAAGGCACT/3MGBEc/− 3′.

### Lung cell transfection

A549 cells, adenocarcinomic human alveolar basal epithelial cells, were purchased from American Type Culture Collection and cultured in DMEM supplemented with 10% FBS, L-Glutamine (2 mM), penicillin (100 U/ml) and streptomycin (100 μg/ml) (Sigma-Aldrich, Milan Italy) in an atmosphere of 5% CO_2_ at 37 °C. Cell transfection was performed following manufacturer’s instructions (Transit kit, Mirus Bio Inc., USA). Caspase-4 sequence (NM_001225.3) was encoded into pcDNA3.1 + C-6His and used for cell transfection at the concentration of 50 ng/ml (Genscript Inc., Netherlands). In particular, four different pcDNA plasmids were used according to the caspase-4 mRNA sequence encoded: 1. pcDNA-1 (PC-1): sequence from nucleotide (nt) 74–1205; 2. pcDNA-2 (PC-2): sequence from nt 74–810; 3. pcDNA-3 (PC-3): sequence from nt 348–1205; 4. pcDNA-4 (PC-4): sequence from nt 423–886. Empty vector was used as negative control.

### Cell proliferation assay

Transfected and non-transfected A549 cells were previously marked by using carboxyfluorescein diacetate succinimidyl ester (CFSE; 5 μM; Molecular Probes, Invitrogen) to perform proliferation assay. CFSE flow cytometry data was analyzed by means of ModFit4.0 software (BD Pharmingen). In some experiments, non-transfected A549 cells were treated with human recombinant (ImmunePharma srl., Italy) of the large subunit of caspase-4 (100 ng/ml) and co-cultered with peripheral blood mononuclear cells (PBMCs) obtained by NSCLC patients (ratio 1:5). PBMCs were isolated by means of Ficoll’s protocol as previously reported (Molino et al., 2019).

In another type of experiments, transfected A549 cells were treated with specific pharmacological inhibitors, such as anti-EGFR (10 μg/ml, AbCam, UK, FTI-276 (5 μg/ml, k-Ras inhibitor, Sigma Aldrich, Rome, Italy), SAHA (5 μg/ml, histone deacetilase, HDAC, inhibitor, Sigma Aldrich, Rome, Italy), 5-AZA (5 μg/ml, DNA methylase inhibitor, Sigma Aldrich, Rome, Italy) and rapamycin (1 μg/ml, mTOR inhibitor, Sigma Aldrich, Rome, Italy).

### Statistical analysis

Data are reported as median ± percentile range and represented as violin plots. Statistical differences were assessed with TWO-WAY or ONE-WAY Analysis of variance (ANOVA) followed by multiple comparison post-tests as appropriate. Percent survival was estimated by means of Kaplan-Meier method and compared with a non-parametric log-rank test. Percent survival was calculated from the time of surgical resection. The survival rate was calculated for 73 patients which tissue-derived biological samples could be tested by means of the ELISA kit. *p* values less than 0.05 were considered significant.

## Results

### Caspase-4 is involved in non-small cell lung Cancer

**Fig. 1:**
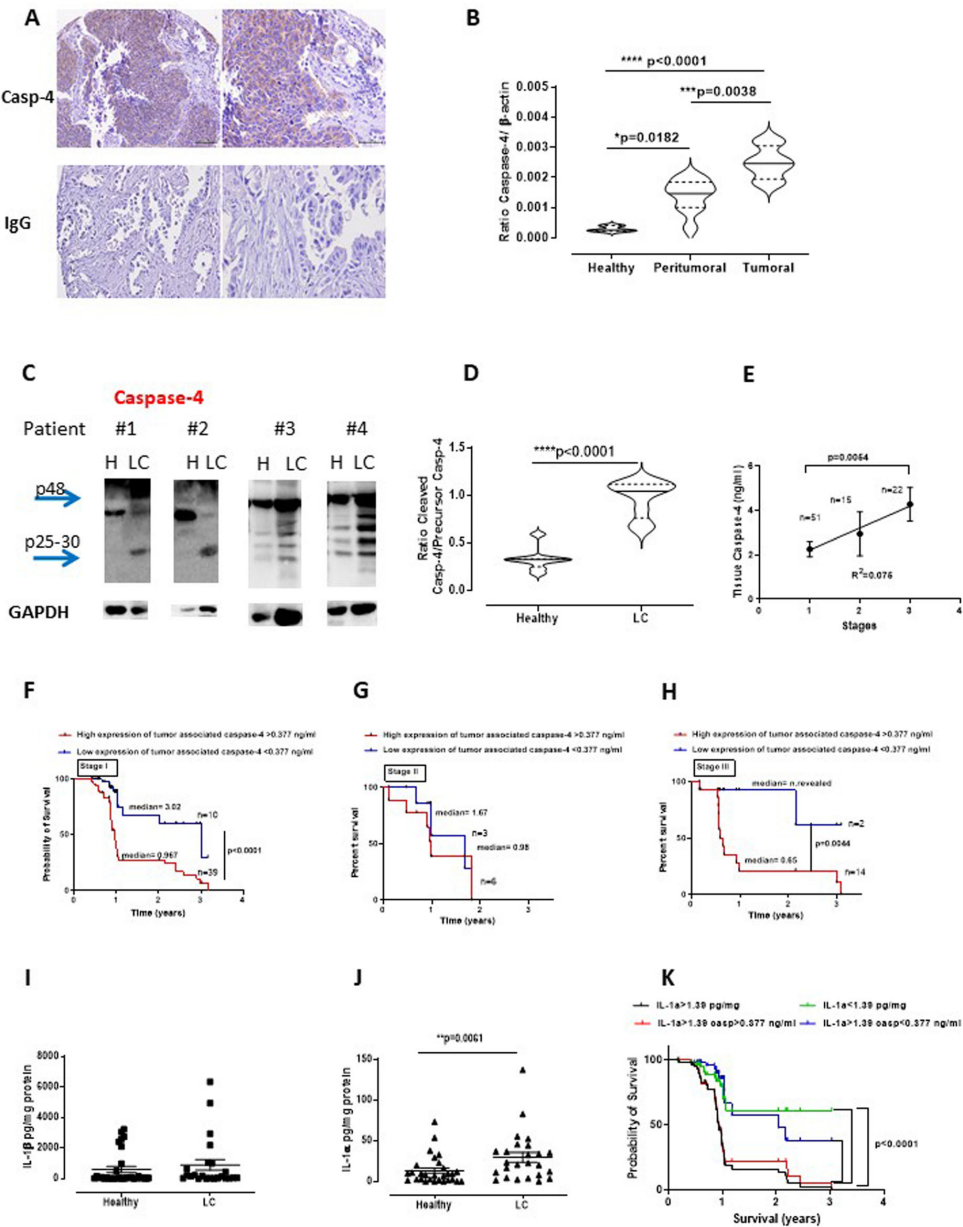
Caspase-4 positive NSCLC patients show poor survival rate. Higher levels of tumor caspase-4 were detected histologically (*n =* 89) (**a**), by means of RT-PCR (*n =* 8) (**b**) and western blotting (*n* = 9) (**c**). Representative blots are shown for immunohistochemistry (**a**). Healthy tissues represent the non-cancerous tissues obtained after surgical resection of tumor areas. **d** Quantitative analysis of western blotting data. The levels of caspase-4 were related to the TNM stage (**e**) of NSCLC patients. **f** Survival rate of stage I NSCLC patients who presented > or < levels of caspase-4 according to the cut-off = 377 pg/ml; **g** Survival rate of stage II NSCLC patients who presented > or < levels of caspase-4 according to the cut-off = 0.377 ng/ml; **h** Survival rate of stage III NSCLC patients who presented > or < levels of caspase-4 according to the cut-off = 377 pg/ml. Levels of IL-1β (**i**) and IL-1α (**j**) in lung homogenates deriving by non-cancerous (healthy) and cancerous (LC) tissues. **k** Survival rate of NSCLC patients according to IL-1α and caspase-4 levels (*n =* 61). Data are expressed as median ± quartile range and represented as violin plots. One Way ANOVA followed by Bonferroni’s post-test was applied to Fig. B. Mann Whitney test was applied to Fig. D, I and J; Two-Way ANOVA was applied to Fig. E. Log-rank Mantel-Cox and Wilcoxon test were performed to statistically analyze the survival rate between the four groups

Recently, we demonstrated that NSCLC patients had higher circulating levels of caspase-4 than healthy subjects [12]. In this study we found that NSCLC tissues had a positive histological score of caspase-4 which was higher in the tumor masses than in non-cancerous tissues (Fig. 1a). In particular, as already observed by using a different technical approach [12], more than 70% of patients presented tissue positivity to caspase-4 (Table 1). Similarly, transcriptional levels of CASP4 mRNA were higher in the tumor mass than in healthy (non-cancerous) tissues of NSCLC patients (Fig. 1b). To note, peritumoral areas had intermediate levels of caspase-4 mRNA compared to healthy (non-cancerous) and tumoral areas (Fig. 1b). These data were confirmed by means of western blotting (Fig. 1c and d), which showed that lung cancer tissues were characterized by the presence of the cleaved form of casapse-4 (25–30 kDa) than healthy tissues. Based on these data, we hypothesized that the presence of caspase-4 could be related to the TNM stage of the pathology. Interestingly, the cleaved form of caspase-4, herein identified as tumor-associated caspase-4, statistically increased from stage I up to stage III (Fig. 1e, stage I to III). To correlate the malignancy stage to the levels of tissue-associated caspase-4, we chose a cut-off value of the protein, as already reported [12] and correlated it to the survival rate. The survival rate of NSCLC patients at stage I who presented higher levels (> 377 pg/ml) of tissue-associated caspase-4 was lower (median = 0.967 years) than patients at stage I who presented lower (< 377 pg/ml) levels (median = 3.02 years) (Fig. 1f). Similarly, NSCLC patients at stage II had lower median survival rate when tumor-associated caspase-4 levels were higher than 377 pg/ml (Fig. 1g). However, in this last case we were not able to reach statistical significance due to the low number of patients. Moreover, stage III NSCLC patients had worse survival rate (median = 0.65 years) when tumor-associated caspase-4 was > 377 pg/ml compared to patients who had lower levels (median = not revealed; *n* = 2) (Fig. 1h). Again, it has to be pointed out that the number of patients at stage III were low.

Caspase-4 in humans and caspase-11 in mice have been widely associated to the non-canonical pathway of the inflammasome [8, 9]. Therefore, we went on by analyzing the levels of tissue IL-1β and IL-1α, two cytokines derived by the inflammasome activation [8, 11, 15]. We found that, despite what reported in literature [16, 17], NSCLC patients did not have differential levels of tissue IL-1β (Fig. 1i). Instead, we found that IL-1α was significantly increased in the tumor tissues than non-cancerous tissues (Fig. 1j). Because we already demonstrated a correlation between IL-1α and caspase-4 [4, 18], we found that patients who presented levels of IL-1α > 1.39 pg/mg protein (cut-off value chosen according to ROC analysis) in the tissue (black line, Fig. 1k) had similar survival rate as patients who had IL-1α > 1.39 pg/mg protein and caspase-4 > 377 pg/ml (Fig. 1k, red line), compared to patients who had lower levels of both caspase-4 and IL-1α (Fig. 1k, green and blue lines).

Taken together these data suggest that tumor tissues are characterized by high levels of caspase-4 and IL-1α which are associated to lower survival rate of NSCLC patients.

### Higher levels of tumor-associated caspase-4 are present in the lung of NSCLC patients with poor survival rate

**Fig. 2:**
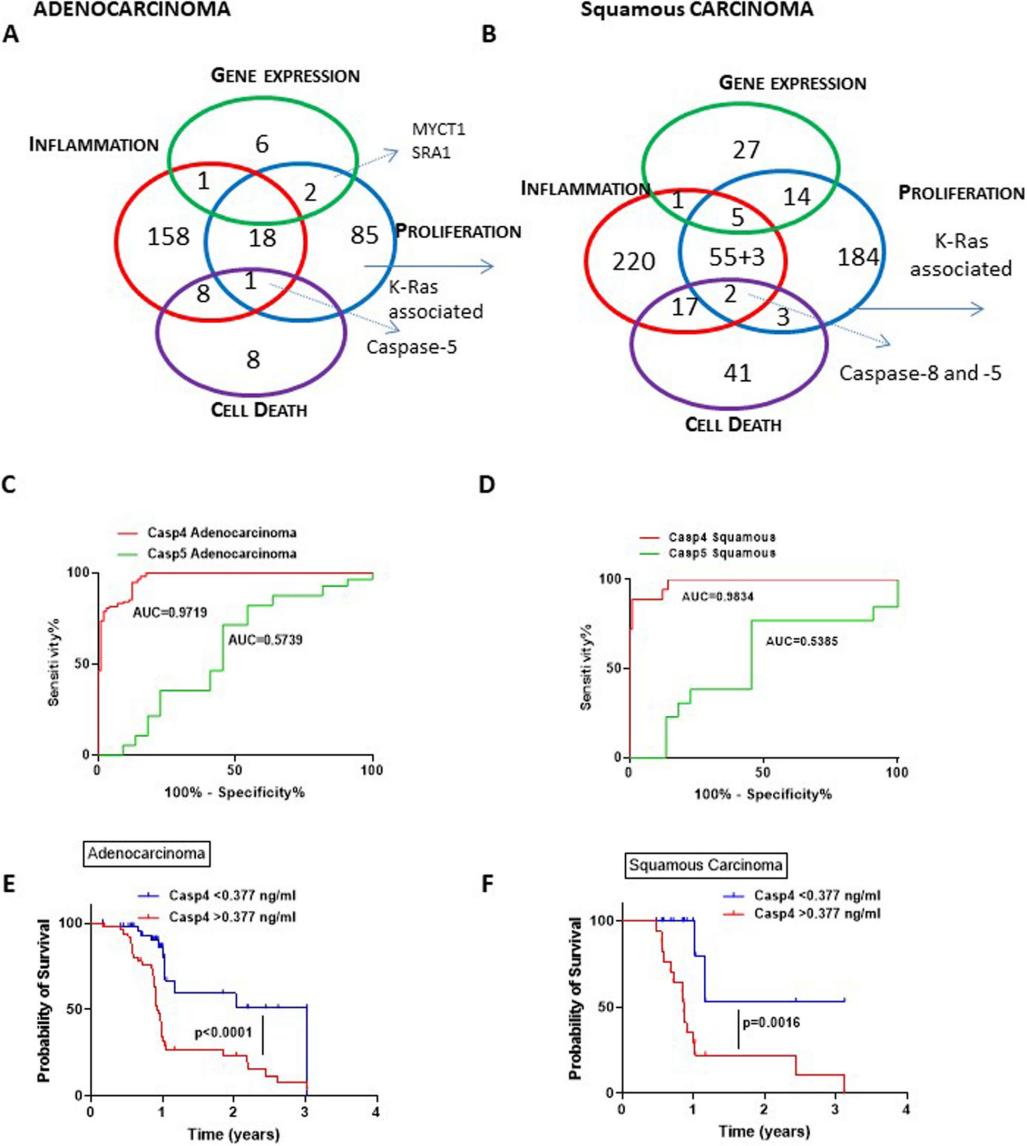
Poor survival rate of adenocarcinoma and squamous lung cancer patients according to the levels of tumor-tissue caspase-4. **a** and **b** Venn’s diagrams obtained by the analysis of genomic alteration on CASP4 (mutation or amplification or higher gene copy number) correlated to other genes involved cell death, inflammation, gene expression and cell proliferation in adenocarcinoma (**a**) and squamous (**b**) lung cancer patients by using a public database (www.cbioportal.org). Common genes were related to KRAS, CMYC and CASP5. ROC analyses for caspase-4 and caspase-5 analyzed in samples obtained by adenocarcinoma (*n* = 66) (**c**) and squamous (*n* = 13) (**d**) patients. Tumor levels of caspase-4 and caspase-5 analysed by means of ELISA were correlated to the available survival rate of (**e**) adenocarcinoma (*n* = 53) and (**d**) squamous (*n* = 17) NSCLC patients. Median survival of adenocarcinoma patients with high of caspase-4 expression (**e**, red line) was 0.925 years vs median survival of patients with lower expression (blue line, 3.028 years). Median survival of squamous carcinoma patients with high expression of caspase-4 (**f**, red line) was 0.864 years vs median survival of patients with lower expression (blue line, not defined). Log-rank Mantel-Cox and Wilcoxon test were performed to statistically analyse the survival rate between the groups

To better understand the role of caspase-4 in lung cancer, we took advantage of a public database (www.cbioportal.org) where we analyzed what already reported in the literature regarding this enzyme. We found that 2% of patients in the public database presented a genomic alteration of CASP4 gene, intended as mutation or amplification or higher gene copy number. In this pattern we analyzed positive correlation between CASP4 and other genes according to Spearman correlation coefficient > 0.25 taking into consideration both adenocarcinoma and squamous carcinoma patients reported in the database. In adenocarcinoma patients, CASP4 positively correlated to 9 genes involved in gene expression (i.e. C-MYCT1, SRA1, GTF2B, EEF1A1, SP110, CNOT8), 186 genes involved in inflammation (i.e. CASP1–5, CARD16, CD63, HLA, IRF1, TLRs), 106 genes involved in cell proliferation (i.e. C-MYCT1, SRA1, RHOA, CDK7, RAB32, CD74, RRAS) and 17 genes involved in cell death (i.e. CASP5, ANXA5, BAK1, RIPK2, MLKL) (Fig. 2a). Similarly, in squamous carcinoma CASP4 positively correlated to 47 genes involved in gene expression (i.e. CREM, BATF, BZW1, HDAC9, STAT1–4), 303 genes involved in inflammation (i.e. AIM2, CASP5, CARD6–16–17-19, CCLs, ICOS, TLRs), 266 genes involved in cell proliferation (i.e. FAM107B, FGF7, IRF1–8-9, RASGRP3) and 63 genes involved in cell death (i.e. CASP8, ANXA3–5, FAS, GZMs, RIPK3) (Fig. 2b). Among these, we found that caspase-5 and K-Ras correlated to CASP4 in both histotypes (Fig. 2a and b, common areas in the Venn diagram).

In order to understand the impact of caspase-5 in NSCLC, we used a caspase-5 antibody to test via an ELISA assay the presence of this enzyme in tumor tissues compared to caspase-4, which was detected by means of ELISA by using a specific antibody, different from what is commercially available, actually under patent (RM2014A000080 and PCT/IB2015/051262). ROC analyses showed that the detection of caspase-4 in lung tumor masses was an excellent diagnostic tool for both adenocarcinoma (Fig. 2c, red line) and squamous carcinoma (Fig. 2d, red line) compared to caspase-5 (Fig. 2c and d, green line). These data are in support to what already published [12], but further highlights that caspase-5, differently than caspase-4, is not associated to the cancerous pattern as it is also present in non-cancerous tissues. This effect was better described by the value of the AUC which was 0.5739 in adenocarcinoma (Fig. 2c, green line) and 0.5385 in squamous carcinoma tissues (Fig. 2d, green line). In support, adenocarcinoma patients who had tumor-associated levels of caspase-4 higher than 377 pg/ml (*n* = 42/53, 79,3%) survived less (median = 0.925 years) than patients with lower levels (*n* = 11/53, 20,7%; median survival =3.03 years) (Fig. 2e). Similar scenario was observed for squamous carcinoma patients. Higher tumor-associated caspase-4 (*n* = 15, 88.2%) was associated to lower survival rate (median = 0.86 years) than those who had lower levels (*n* = 2, 11.8%; median = undefined) (Fig. 2f). These data, despite the low number of patients in Fig. 2f, most likely imply that tumor-associated caspase-4, but not caspase-5, is correlated to lung carcinogenesis.

### Higher levels of tumor-associated caspase-4 are present in the lung of PD-L1 negative NSCLC patients

**Fig. 3:**
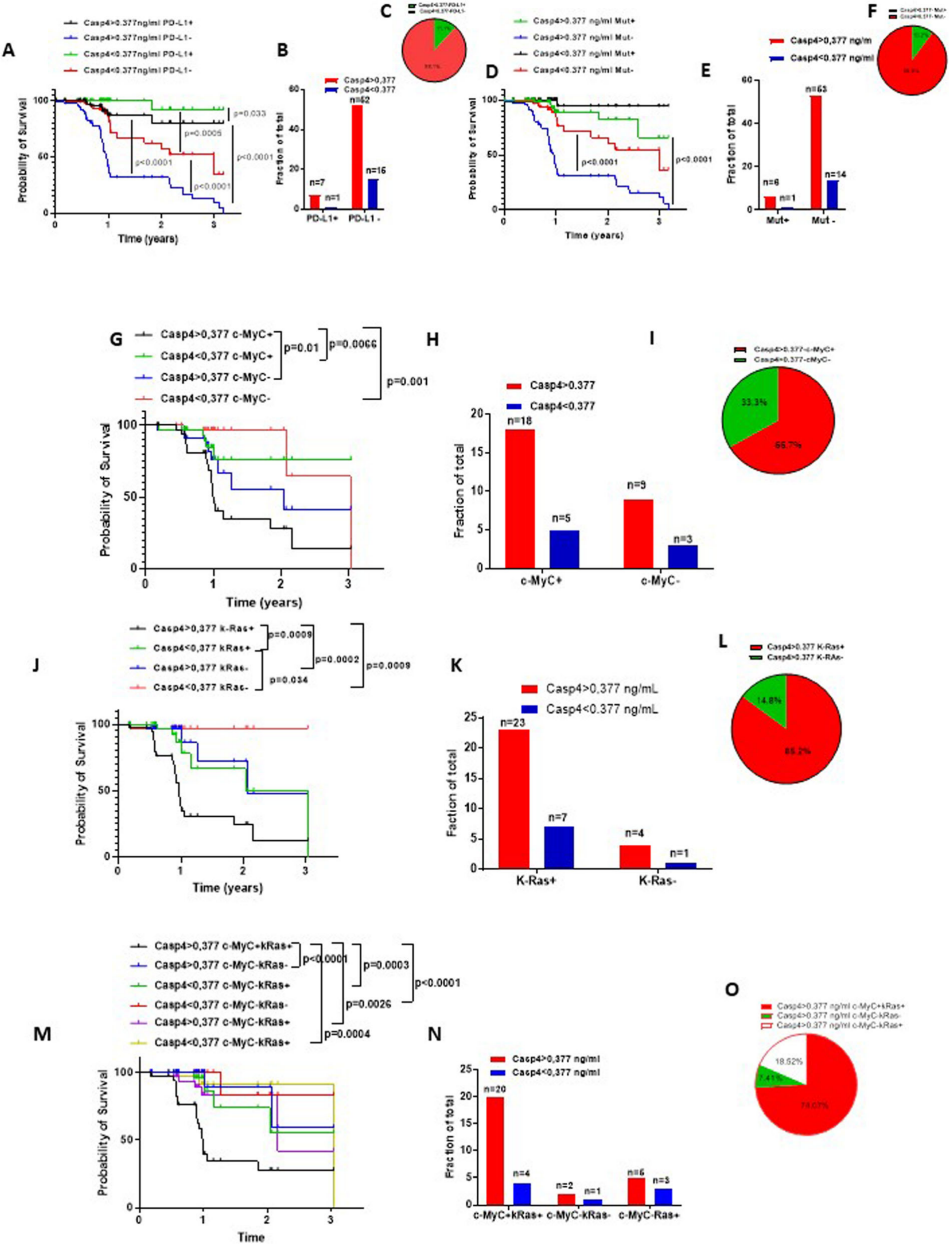
Triple positive Caspase-4, K-Ras and c-Myc NSCLC patients have poor survival rate. NSCLC patients were stratified according to the levels of caspase-4 related to PD-L1 expression (**a**), gene mutation (i.e. EGFR mutation, ALK or MET or ROS1 translocation) (**b**), or c-Myc overexpression (**g, m**) and K-Ras mutations (**j, m**). In addition, fraction of total was analysed (**b, e, h, k, n**) to better highlight the fraction of analysed patients according to the levels of tumor tissue caspase-4 based on a cut-off (377 pg/ml) obtained by ROC analysis. Furthermore, the percentage of caspase-4 positive patients were related to PD-L1 expression (**c**), gene mutation (i.e. EGFR mutation, ALK or MET or ROS1 translocation) (**f**), or c-Myc overexpression (**i, o**) and K-Ras mutations (**l, o**). Log-rank Mantel-Cox and Wilcoxon test were performed to statistically analyse the survival rate between the groups

NSCLC patients could be classified according to mutations and PD-L1 positivity [2]. Therefore, we stratified NSCLC patients as PD-L1 positive vs PD-L1 negative according to the levels of tumor-associated caspase-4. We found that NSCLC patients who had higher levels of tumor-associated caspase-4 (> 377 pg/ml, cut-off) but were negative for PD-L1 had lower survival rate (median survival = 0.96 years; % of survival rate at 1 year≈30%) (Fig. 3a, blue line) (Fig. 3b, *n* = 52/75, 69.3%) than patients (9.3%) who had higher levels of tumor-associated caspase-4 but PD-L1 positive (median survival = undefined; % survival rate at 1 year≈80%) (Fig. 3a, black line; Fig. 3b, *n* = 7/75, 11.1%). However, patients with higher levels of tumor-associated caspase-4 but positive for PD-L1 (Fig. 3a, black line) showed lower survival rate (*p* = 0.033) than patients with low caspase-4 but positive for PD-L1 (Fig. 3a, green line, *n* = 1/75, 1.3%, median survival = undefined). Nevertheless, it has to be noted that there was a group of patients who were PD-L1 negative and presented lower tumor-associated caspase-4 (Fig. 3a, red line; Fig. 3b, *n* = 15/75, 20%, % survival at 1 year = 85%) whose median survival rate was of 2.98 years. These latter group survived more than patients with high levels of caspase-4 and PD-L1 negative (Fig. 3a, blue line), survival rate that was still lower than patients who had PD-L1 positivity and high caspase-4 (Fig. 3a, black line) and PD-L1 positivity and low caspase-4 (Fig. 3a, green line). These data imply that a group of patients (Fig. 3b, *n =* 15/75, 20%), whose survival is still low, is independent from caspase-4 and PD-L1. Though, it is noteworthy that the survival of this group at 1 year is ≈80% (Fig. 3a, red line) compared to the group with high caspase-4 and PD-L1 negative (Fig. 3a, blue line), who instead, presented a survival rate at 1 year of ≈30%. Based on these analyses, among patients who presented with high levels of caspase-4 (Fig. 3b, red bar), those who were negative for PD-L1 represented 88.1% (Fig. 3c) compared to PD-L1 positive patients (Fig. 3c, 11.9%), implying a further stratification of NSCLC patients. Moreover, according to chi squared test, caspase-4 and PD-L1 were independent biomarkers as also observed in Fig. 3b and c where caspase-4 positive and PD-L1 negative patients were the majority of patients in our database.

### Higher levels of tumor-associated caspase-4 are present in the lung of K-Ras and c-MyC-mutated NSCLC patients

Another important issue for NSCLC patients is about gene mutation/s [2]. In this latter case, we stratified patients who presented EGFR mutation or MET, ROS1 or ALK translocation, herein defined as mutated (Mut^+^), and compared them to the levels of tumor-associated caspase-4. We found that NSCLC patients who had high levels of tumor-associated caspase-4 (> 377 pg/ml) and did not have any gene mutation (Mut^−^) (Fig. 3d, blue line) had lower survival rate (median survival = 0.95 years; % of survival rate at 1 year≈35%) (Fig. 3e, *n* = 53/74, 71.6%) than patients who had higher levels of tumor-associated caspase-4 and were Mut^+^ (survival at 1 year≈80%) (Fig. 3d, green line; Fig. 3e, *n* = 6/74, 8.1%). As observed before, there was a group of patients who were Mut^−^ and had lower tumor-associated caspase-4 (Fig. 3d, red line; Fig. 3e, *n* = 14/74, 18.9%). This group of patients had a median survival rate of 2.98 years and however, had higher survival rate than patients with higher levels of caspase-4 and Mut^−^ (Fig. 3d, blue line). In addition, the survival rate of patients who had Mut^+^ and high caspase-4 (Fig. 3d, green line) was still lower than Mut^+^ and low caspase-4 (Fig. 3d, black line), implying a group of patients (Fig. 3e, *n =* 14, 18.9%) where the survival is still low without any relationship with caspase-4. The survival rate of this group at 1 year was of ≈85% compared to the group with high caspase-4 but Mut^−^ (Fig. 3d, blue line), that instead presented survival rate at 1 year of ≈35%. Based on these analyses, among caspase-4 positive patients (Fig. 3f, red bar) those who were Mut^−^ represented 89.8% (Fig. 3f) compared to Mut + patients (Fig. 3f, 10.2%). Moreover, according to chi squared test, caspase-4 and genetic alterations were independent biomarkers as also observed in Fig. 3e and f where caspase-4 positive and Mut^−^ patients were the majority of patients in the database.

As shown in the Venn diagram (Fig. 2a-b), caspase-4 was associated to genes involved in cell proliferation, such as K-Ras and c-MyC, well-known to be involved in lung carcinogenesis [2, 19, 20]. Therefore, we analyzed both K-Ras mutations (G12C, G12D and G12V) and c-MyC expression related to caspase-4 levels in our experimental human samples. We found that NSCLC patients with high levels of tumor-associated caspase-4 and c-MyC positive (c-MyC^+^) (Fig. 3g, black line) had a median survival of 1 year, lower than patients who had lower levels of caspase-4 and c-MyC^+^ (Fig. 3g, green line; median survival = undefined). Patients who presented no positivity to both caspase-4 and c-MyC had longer survival than the other groups (Fig. 3g, red line, median survival = 3.028). Interestingly, patients with high levels of tumor-associated caspase-4 but who were c-MyC negative (c-MyC^−^) still had lower survival rate (median survival = 2.036 years) (Fig. 3g, blue line), although it was higher than patients positive for both Caspase-4 and c-MyC (Fig. 3g, black line). The survival rate at 1 year was of 50% for Caspase-4-c-MyC^+^ patients, of ≈95% for Caspase-4-c-MyC^−^ and of ≈85% for Caspase-4 negative c-MyC^+^ and Caspase-4 positive c-MyC^−^ patients (Fig. 3g). Based on these analyses, patients who presented with high levels of caspase-4 (Fig. 3h, red bars) and c-MyC+ were 66.7% in our database (Fig. 3i), implying that caspase-4 and c-MyC gene overexpression were strictly associated.

Similarly, we stratified patients according to the positivity to mutated K-Ras (G12C, G12D and G12V). We considered as positive those patients who presented at least one of the three mutations. Very interestingly, the survival rate of caspase-4^+^ and K-Ras^+^ patients was significantly reduced (0.97 years) (Fig. 3j, black line) compared to patients who were caspase-4^−^ and K-Ras^−^ (Fig. 3j, red line). Moreover, both groups of patients who were caspase-4^+^ and K-Ras^−^ or caspase-4^−^ and K-Ras^+^ had median survival rate of 2.07 and 3.028 years, respectively, further highlighting the relevance of caspase-4 (Fig. 3j and k, green and blue line). To date, we found that the majority of patients were caspase-4 + and K-Ras + (Fig. 3l, 85.2%), compared to caspase-4 + but K-Ras – patients (Fig. 3l, 14.8%).

In addition, we stratified patients as caspase-4, c-MyC and K-Ras triple positive. Patients who were positive for the three targets had a survival rate of 0.98 years (Fig. 3m, black line), similar to that observed for caspase-4 and K-Ras + (Fig. 3j, black line, median survival = 0.97 years) and c-MyC+ (Fig. 3g, black line, median survival = 1 year) patients. This group of patients represented ≈74.1% (Fig. 3n-o). Nevertheless, patients who were solely positive for caspase-4 still represented ≈7.4% of the study population (Fig. 3n-o).

These data highlight that caspase-4 collaborates with c-MyC and K-Ras leading to a bad prognosis of NSCLC patients.

### Caspase-11 facilitates lung tumor progression in mice

**Fig. 4:**
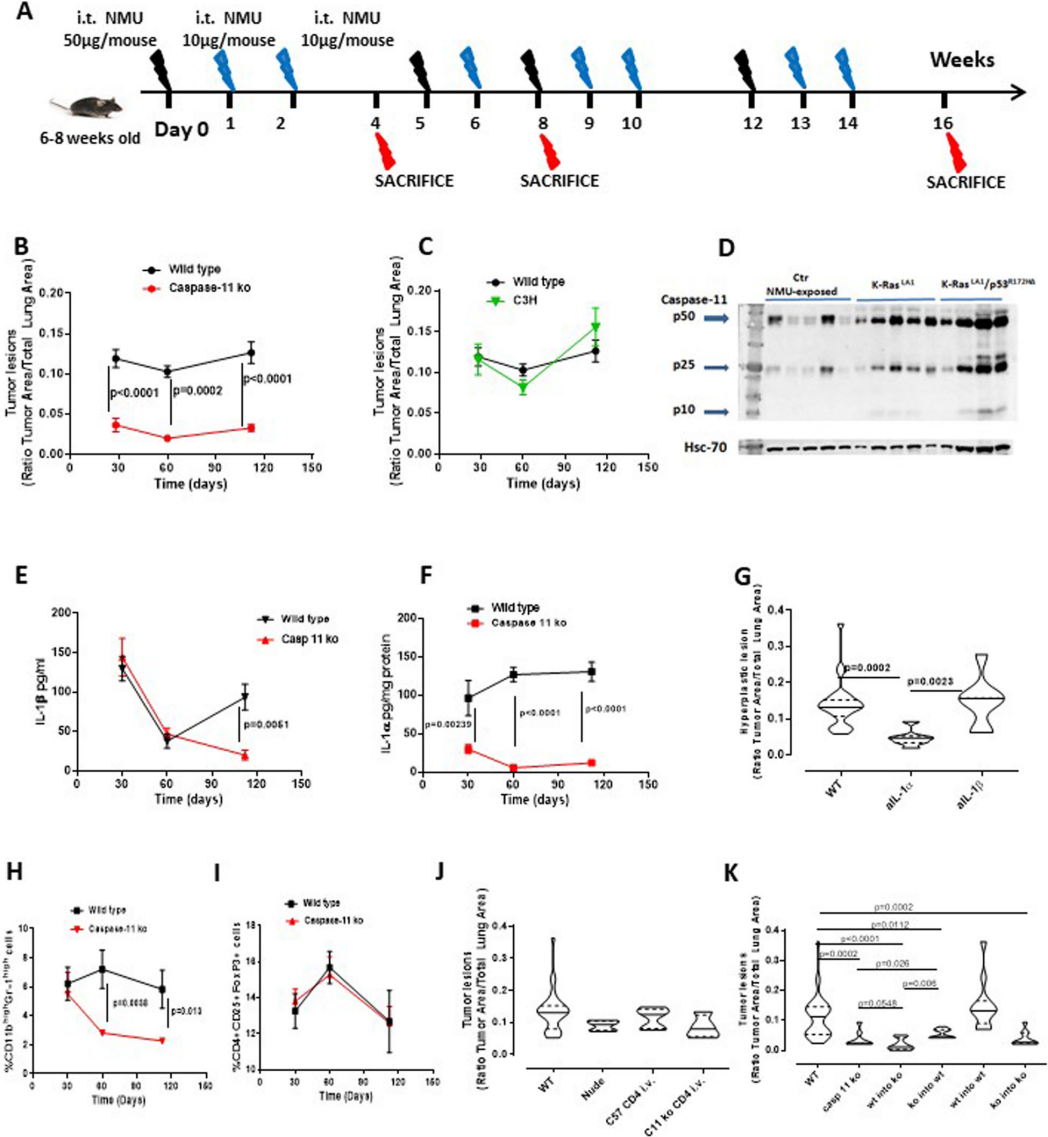
Caspase-11 is involved in tumor progression in mice. **a** Mouse model of NMU-induced carcinogenesis. **b** Wild type mice (*n* = 23) showed higher tumor lesions than caspase-11 ko (*n* = 12) mice in a time-dependent manner. **c** C3H mice (*n* = 14) did not show any difference in terms of tumor areas than wild type mice. **d** Lung tissues of K-Ras ^LA1^ (*n* = 10) and K-Ras ^LA1^p53^R172HB^ (*n =* 10) had similar levels of cleaved caspase-11 as wild type NMU-treated C57Bl/6 mice. **e** BAL levels of IL-1β in caspase-11 ko mice (*n =* 12) were solely reduced at longer time points compared to wild type mice (*n =* 23); instead, **f** IL-1α levels in lung homogenates were significantly reduced at all time points in caspase-11 ko mice (*n =* 12) compared to wild type mice (*n =* 23) subjected to NMU. **g** The neutralization of IL-1α by means of a monoclonal antibody significantly reduced tumor hyperplastic cells in the lung of NMU-treated C57BL/6 mice. Lung immune infiltration was characterized by MDSC (**h**), but not by Treg (**i**) in NMU-treated wild type but not in caspase-11 ko mice. **j** No differences in tumor area was observed in NMU-treated mice or in mice who were subjected to the adoptive transfer (*n =* 10) of CD4+ T cells obtained by C57Bl/6 or caspase-11 ko mice. **k** Bone marrow transplantation experiments showed that wt into ko mice had similar tumor area as caspase-11 ko mice treated with NMU (*n =* 12). Data are expressed as mean ± SEM or as median ± quartile range (violin plots). Two-Way ANOVA followed by Sidak’s multiple comparison test was applied to Fig. B, C, E, F, H, I. The statistical analysis performed for Fig. B and C considered the comparison among and within the groups according to two variables, tumor lesion vs time; instead the statistical analysis for Fig. E and F was performed according to the variation of the amount of cytokine released (expressed as pg/ml) vs time, as well as in Fig. H and I the variables were percentage of cells vs time. In contrast, One Way ANOVA followed by Bonferroni’s post test was applied to Fig. G, J, K, as the statistical test was related to the variation within and among the groups according the only one variable

Experimental biological samples showed that capase-4 is related to poor survival of NSCLC patients. To better investigate the molecular mechanism, we went on by taking advantage of a mouse model of adenocarcinoma [13]. C57Bl/6 mice (wild type) and Caspase-11 knockout (Caspase 11-ko) were i.t. injected with NMU at week 1–2-3, 8–9-10, 12–13-14 and sacrificed at week 16 (Fig. 4a). Very importantly and in support to our previous human data, caspase-11 ko mice robustly developed lower lung tumor lesions than wild type mice (Fig. 4b). Because LPS, a TLR4 ligand, was supposed to be able to induce caspase-11 activation via a second signal model [21], to rule out the role of TLR4, we used C3H mice who are defective in TLR4 signalling [22, 23]. Surprisingly, C3H mice had similar lung tumor lesions as C57Bl/6 wild type mice (Fig. 4c), implying that caspase-11-related pro-tumor activity is directly involved in lung tumor progression in mice. Therefore, because we previously demonstrated that the majority of NSCLC patients were caspase-4 and K-Ras^+^, we used lung tissues of K-Ras ^LA1^ and K-Ras ^LA1^p53^R172HB^ mice who were previously described by Prof. Quaglino’s laboratory [24] as a lung adenocarcinoma model that shows lung tumor lesions starting from 10 weeks of age. K-Ras^LA1^ mice, similar to C57Bl/6 mice injected with NMU, were characterized by cleaved caspase-11 (p25 fragment) (Fig. 4d). Moreover, K-Ras ^LA1^p53^R172HB^ mice, who were characterized by mutated K-Ras and p53 and high lung tumor lesions [24], showed not only p25 but also p10 fragment (Fig. 4d). Noteworthy, the precursor form of caspase-11 was higher expressed in these latter mice compared to NMU-injected C57Bl/6, control (Ctr) mice (Fig. 4d), implying that not only K-Ras mutation, but also p53 genetic alteration could be implied in caspase-11-related pro-tumor activity in mice.

Caspase-11 was described as involved in the non-canonical inflammasome pathway and associated to IL-1-like cytokine release [10, 11, 13, 18, 21]. We found that IL-1β was reduced in the BAL of caspase-11 ko mice at longer time point (16 weeks) (Fig. 4e) compared to wild type mice. Instead, as also observed in human samples (Fig. 1j), IL-1α was significantly higher in the lung of wild type mice than caspase-11 ko mice (Fig. 4f) at all-time points (30–56-116 days), implying a major role of IL-1α. To better understand the relevance of IL-1α vs IL-1β in our experimental conditions, we injected mice with a monoclonal antibody against IL-1α or IL-1β. The neutralization of IL-1α significantly reduced the number of hyperplastic cells in the lung of NMU-injected C57Bl/6 mice (Fig. 4g, aIL-1α) compared to the control group (CTR) and mice who were similarly treated with a neutralizing antibody against IL-1β. The IgG isotype control group showed similar hyperplastic cell number (0.133 ± 0.07) as CTR mice (0.139 ± 0.06).

Tumor immunosuppression has been widely described as pivotal for cancer progression [11, 13, 25]. Therefore, we moved on to analyze the immune microenvironment in the lung of NMU-treated tumour-bearing mice. The percentage of myeloid-derived suppressor cells (MDSCs; identified as CD11b^+^Gr-1^high^) were significantly reduced in the lungs of NMU-treated caspase-11 ko mice compared with wild type (Fig. 4h). In contrast, the percentage of T regulatory cells (Treg: identified as CD4^+^CD25^+^FoxP3^+^ cells) was not altered in caspase-11 ko mice compared to wild type (Fig. 4i), implying that the adaptive immunity was not affected by the genetic absence of caspase-11. Therefore, we injected NMU-treated Nude mice with isolated CD4^+^ cells obtained from wild type or caspase-11 ko mice (Fig. 4a). We found that NMU-treated Nude mice, although with a tendency to reduction, not statistically significant, had similar lung tumor lesions as wild type (C57Bl/6) mice (Fig. 4j). Interestingly, neither the adoptive transfer of wild type CD4^+^ T cells nor the adoptive transfer of caspase-11 ko CD4^+^ T cells altered lung tumor lesions (Fig. 4j), implying that caspase-11 has no relevance in the adaptive immunity. In contrast, bone marrow transplantation experiments showed that caspase-11 ko mice receiving wild type bone marrow-derived cells (Fig. 4k) had the lowest number of lung tumor lesions compared to wild type mice receiving ko bone marrow-derived cells (Fig. 4k; ko into wt vs wt) and wild type lung tumor-bearing mice (Fig. 4k; wt into ko vs casp 11 ko). To note, NMU-treated caspase-11 ko mice receiving wild type bone marrow-derived cells had similar levels of tumor lesions as NMU-treated caspase-11 ko mice (Fig. 4k, wt into ko vs casp 11 ko), implying that the structural caspase-11 is involved in lung cancer progression in mice. Nevertheless, NMU-treated wild type mice receiving ko bone marrow-derived cells (Fig. 4k; ko into wt) still had lower lung tumor lesions compared to NMU-treated wt mice, although the tumor lesions was higher than those in caspase-11 ko mice receiving wild type bone marrow-derived cells (Fig. 4k; ko into wt vs wt into ko). Taken altogether, these data suggest that caspase-11 is involved in lung tumor progression in mice influencing the innate immunity most likely via IL-1α.

### Large subunit of Caspase-4 facilitates tumor cell proliferation

**Fig. 5:**
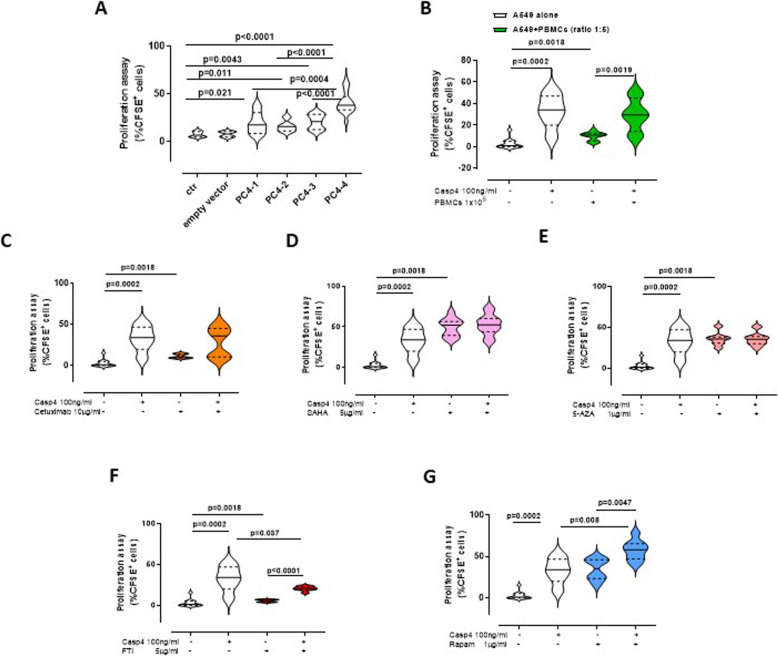
Large subunit of caspase-4 facilitates tumor cell proliferation. A549 cells were transfected with PC4–1, PC4–2, PC4–3 and PC4–4 as explained in Materials and Method section. **a** Cells transfected with PC4–4 (the sole large subunit of caspase-4) showed higher CFSE positive cells than the other groups (*n =* 15). **b** Co-culture of A549 cells and PBMCs (ratio 1:5) showed similar proliferation expressed as %CFSE+ cells than A549 alone treated with the recombinant large subunit of capase-4. The neutralization of EGFR by means of cetuximab (**c**), inhibition of HDAC by means of SAHA (**d**), inhibition of methyltransferase (**e**) and mTOR (**g**) did not alter cell proliferation as instead observed when the inhibitor of k-RAS (FTI-276) was added (**f**). Experiments were performed *n =* 15 in duplicate. Data are expressed as median ± quartile range and represented as violin plots. One-Way ANOVA followed by Bonferroni’s post test was applied

The above data imply that caspase-4 in the structural cell component of lung tissues can favor tumor formation and progression. Therefore, to go inside the molecular mechanism we transfected A549 cells, mimicking lung epithelial cells, with viral vectors to express the long structure of caspase-4 (mRNA: 74–1205 nucleotides, nt, CARD+LARGE+SMALL subunit, PC4–1), the structure of the protein with CARD+LARGE subunit (mRNA: 74–810 nt, PC4–2), the structure of the protein with LARGE+SMALL subunit (mRNA: 348–1205 nt, PC4–3) and the structure of the protein with the sole LARGE subunit (mRNA: 423–886 nt, PC4–4). Lung epithelial cells transfected with PC4–1, PC4–2 and PC4–3 showed higher proliferation rate than control and empty vector-transfected cells (Fig. 5a). Because the three vectors had a common nucleotidic sequence which corresponded to the LARGE subunit and because both human and mouse samples showed p25 fragment of caspase-4 and caspase-11 in lung homogenates, we investigated the role of this subunit. Interestingly, we found that cells transfected with PC4–4, which solely contained nucleotides for the LARGE subunit, robustly proliferated compared to control and empty vector transfected cells, but also to PC4–1, PC4–2 and PC4–3 transfected cells (Fig. 5a). Similarly, treatment of cells with the recombinant protein that mimicked the large subunit of caspase-4 increased cell proliferation compared to the control (Fig. 5b; white violin plot). Moreover, the addition of NSCLC patient-derived PBMCs did not alter the proliferation of cells (Fig. 5b, green bars), further highlighting what was previously observed in mice about the role of caspase-11 in lung structural cells.

In order to understand the molecular mechanism underlying caspase-4-induced cell proliferation, we treated cells with cetuximab, a monoclonal antibody against EGFR. Cell proliferation was not altered when cetuximab was added to caspase-4-treated cells (Fig. 5c), implying that EGFR signaling was not involved. Similarly, the inhibition of histone deacetylase (HDAC), highly involved in lung cancer [26], by means of SAHA (Fig. 5d) and of DNA methyltransferase by means of 5-AZA (Fig. 5e) did not modify caspase-4-induced cell proliferation, implying that caspase-4 is unlikely to be involved in epigenetic modulation/s that lead to tumor cell proliferation [27]. In sharp contrast, the inhibition of K-Ras by means of FTI significantly reduced caspase-4-induced cell proliferation (Fig. 5f, red bars). In support, we found that caspase-4 and K-Ras co-immunoprecipitated in lung tumor tissues (Supplementary Fig. 1). To rule out another proliferative pathway, we treated cells with rapamicin, mTOR inhibitor. Again, we did not find an alteration in caspase-4-induced cell proliferation (Fig. 5g, blue bars). These data suggest that the large subunit of caspase-4 induces cell proliferation via K-Ras pathway. In support, we found that caspase-4^+^ and K-Ras mutated patients had lower survival rate than other NSCLC patients, supporting the biochemical analyses.

## Discussion

In this study we found that caspase-4 is correlated to lung carcinogenesis and poor survival rate of NSCLC patients. Herein, caspase-4 could be identified as a novel oncoprotein since 79.3 and 88.2% of adenocarcinoma and squamous NSCLC patients, respectively, stained positive for the protein which pro-tumor activity was reflected in a concerted cooperation with mutated K-Ras and cMyC. Interestingly, a subpopulation of NSCLC patients (20 out of 35 = 57.1%) were triple positive for caspase-4, mutated K-Ras and c-MyC and presented a survival rate of less than 1 year.

Caspase-4 in humans and the analogue murine caspase-11 have been widely described as inflammatory caspases involved in the non-canonical inflammasome pathway in that they are able to sense LPS and lead to the release of IL-1β and IL-18 other than inducing pyroptotic cell death [8, 28], identified by the release of LDH from cells. One limitation of our study, though, is that the endogenous ligand for caspase-4 in lung cancer is still not identified; however, our data demonstrate that caspase-4/caspase-11 are involved in lung carcinogenesis in humans and mice, respectively. This is the first study, to our knowledge, to show the pro-tumorigenic role of caspase-11 in mice and caspase-4 in humans. Caspase-11 activation is regulated via the TLR4/IFN pathway upon TRIF-induced procaspase-11 processing [9]. In this regard, we previously demonstrated that the administration of Poly I:C, a TLR3 ligand, that is solely regulated by TRIF and that leads to IFN type I release, reduced tumor burden in a syngenic lung cancer mouse model [29]. It is noteworthy that Poly I:C-induced reduction of lung tumor in mice was strictly related to the activation of the innate immunity against the tumor. Instead, in this study, we found that caspase-11 is significantly relevant in the structural cell compartment where it is involved in lung carcinogenesis in mice. Indeed, bone marrow transplantation of wild type cells into NMU-treated caspase-11 ko mice, as well as in the case of NMU-treated caspase-11 ko mice, robustly reduced tumor lesions in the lung. Instead, tumor burden of NMU-treated wild type mice that received caspase-11 ko bone marrow cells had higher tumor lesions than NMU-treated caspase-11 ko mice that received wild type bone marrow cells, strengthening what already observed for TAMs which use caspase-11/caspase-1/NLRP3 axis to promote lung tumorigenesis [11].

In support, a very interesting paper by Cheng et al. [26], similarly demonstrated the relevance of the non-hematopoietic caspase-11 in a mouse model of lung injury. However, the latter effect was mediated by TLR4. Instead, in our experimental conditions, we found that TLR4 dysfunctional mice (C3H mice) had a similar tumor burden as wild type mice, implying that caspase-11 was not induced by the non-canonical inflammasome pathway. Most likely, because we found that there was a strict correlation between caspase-4 and IL-1α [4, 12], but not IL-1β, release, we may speculate that caspase-4 activation and IL-1α could be the main orchestrators of lung tumorigenesis in a non-inflammasome-dependent manner in the hematopoietic compartment. It is likely that caspase-4 in the structural cells behaves as an oncoprotein, whereas in the hematopoietic lineage it can allow IL-1α protumorigenic activity. Indeed, the neutralization of IL-1α in NMU-treated mice significantly reduced the levels of tumor areas than control group and, very importantly, NSCLC patients who presented high levels of IL-1α and caspase-4 had lower median survival rate.

Lung tumor-associated caspase-4 was related to tumor cell proliferation, rather than cell death. This effect was correlated to the large subunit of caspase-4. In literature. Caspase-4 and caspase-11 activity are often associated to the release of lactate dehydrogenase, LDH, as a marker of cell death. It is worldwide known that high levels of LDH characterize inflammatory patterns, together with CRP and ERS, which are highly detected in cancer patients. Similarly, in our experimental conditions, we found that all patients positive for tumor-associated caspase-4 had high levels of LDH, which was not a measure of cell death as reported in in vitro assays [27, 30], but of cancer progression [31]. To date, LDH is an enzyme that catalyzes the pyruvate to lactate during anaerobic conditions. The metabolomic profile of cancer patients is well-described as altered in that to promote the accumulation of pyruvate which leads to both the anaerobic (LDH-dependent) and tricyclic acid (TCA) pathway according to the tumor cell metabolic needs [32, 33]. Therefore, tumor-associated as well as circulating caspase-4 [12] suggest that the inflammatory pattern in tumor cells alters the metabolomic profile to favor cell proliferation rather than cell death, as we recently demonstrated [31]. In support, Trinidad et al., proved that the activation of the pyruvate kinase 4, which phosphorylates the pyruvate at the last step of glycolysis, is correlated to the metabolic phenotype of K-Ras in favor of tumor cell proliferation [34]. The oncogenic K-Ras was associated with higher levels of hexokinase 2 (HK2), involved in high-rate metabolism of the glucose in lung cancer-associated TCA according to higher consumption of glutamine [35].

Similarly, various studies have demonstrated that c-MyC-driven tumors display increased glucose uptake and catabolism to lactate and TCA cycle intermediates [36]. In this context we found that 85.2% of NSCLC patients who presented K-Ras mutation and 66.7% of patients who overexpressed c-MyC were positive for tissue caspase-4. The median survival rate of these two subgroups of patients was 0.97 (Fig. 3j, black line) and 1 (Fig. 3g, black line) year. However, our study had a limited number of patients at stage III due to the fact that these patients usually undergo therapeutic treatment without surgical resection. In addition, we found that K-ras^LA1^ and K-ras^LA1/p53R172HΔ^ Tg mice had higher lung levels of the cleaved form of caspase-11 (Fig. 4d), associated to higher tumor lesions developed in double Tg mice as reported by Riccardo et al. [24]. These data together with the in vitro studies (Fig. 5) suggest that caspase-4 underlies K-Ras-mediated cell proliferation. However, it has to be pointed out that Caspase-4+ K-Ras -– (Fig. 3j, blue line; Fig. 3l, 14.8%) or Caspase-4+ cMyC- (Fig. 3g, blue line; Fig. 3i, 33.3%) NSCLC patients still had lower survival rate. In this regard, a recent manuscript demonstrated that caspase-4 can lead to epithelial-mesenchymal transition in lung cancer [37], further highlighting the importance of our discovery on the role of caspase-4 in lung carcinogenesis in humans.

Another important issue is that we found that among PD-L1 negative patients, 88.1% were positive to caspase-4 (Fig. 3c). Similarly, 89.8% of NSCLC patients who did not present EGFR mutation or ALK, ROS1, MET genetic alterations were positive to caspase-4. Key established predictive biomarkers for target therapy include ALK and ROS1 rearrangements, EGFR mutations, BRAF V600E point mutations, and PD-L1 expression levels [2]. The National Comprehensive Cancer Network (NCCN) panel recommends testing for these key established biomarkers in patients with NSCLC to decide for the pharmacological treatment, because effective targeted therapies or immunotherapy are available. In particular, tyrosine kinase inhibitors (TKIs) and monoclonal antibodies against PD-1/PD-L1 axis are becoming the first-line therapeutic options for NSCLC patients positive to these targets. However, patients treated with TKIs develop drug resistance after around 12–15 months of treatment. Similarly, patients treated with anti-PD-1/PD-L1 monoclonal antibodies can develop resistance with various mechanisms [2]. Even worse is the situation of non-mutated NSCLC patients whose sole therapeutic option could be the classical chemotherapy or chemotherapy in addition to anti-PD-L1 antibodies, although the low expression of PD-L1 in tumor tissues. Therefore, here we identified a novel subpopulation of NSCLC patients as caspase-4 positive among which we identified patients as double positive for caspase-4 and K-Ras or c-MyC, and triple positive, identified as caspase-4, K-Ras and c-MyC positive.

## Conclusions

Our study 1. highlights a novel diagnostic and prognostic oncoprotein for a subgroup of NSCLC patients; 2. identifies a subpopulation of NSCLC patients who are caspase-4 positive among non-mutated and K-Ras-mutated and cMyC overexpressing patients; 3. identifies a potential molecular mechanism between caspase-4 and K-Ras/c-MyC in the tumor mass; 4. opens new therapeutic perspectives for patients who can only be cured with the classical chemotherapy since K-Ras and cMyC are still undrugable. Therefore, the identification of caspase-4 as a novel oncoprotein could introduce novelty in the clinical yet unmet needs for NSCLC patients.

## Supplementary Information

**Additional file 1: Fig. S1.** Human caspase-4 co-immunoprecipitated with k-RAS in human tumor samples. Human digested lung tumor samples were analyzed by means of Native-PAGE. The isotype control of the antibody against caspase-4 did not show any aspecific band. Similarly eluted samples did not show any positive band, implying that caspase-4 and K-RAS are bound in human lung tumor samples obtained by NSCLC patients. Experiments were performed three times.

## Data Availability

The datasets used and/or analysed during the current study are available from the corresponding author on reasonable request.

## Abbreviations

NSCLC: Non-small cell lung cancer
PD-1: Programmed death-1
PD-L1: Programmed death ligand-1
EGFR: Epidermal growth factor receptor
ALK: Anaplastic lymphoma kinase
IL-1: Interleukin-1
CRP: C-reactive protein
ESR: Erythrocyte sedimentation rate
HMGB1: High Mobility Group Box 1
NLRP3: Nucleotide-Binding Oligomerization Domain, Leucine Rich Repeat And Pyrin Domain Containing 3
TAMs: Tumor-associated macrophages
ROS1: ROS Proto-Oncogene 1
MET: Met proto-oncogene
ko: Knockout
NMU: N-methyl-N-nitroso-urea
BAL: Broncho-alveolar lavage fluid
TMA: Tissue microarray
CFSE: Carboxyfluorescein diacetate succinimidyl ester
CASP4: Caspase-4 gene
ROC analysis: Receiver operating characteristic analysis

## References

[1] Pinto A (2011). Morello, Sorrentino R. lung cancer and toll-like receptors. Cancer Immunol Immunother.

[2] Terlizzi M, Colarusso C, Pinto A, Sorrentino R (2019). Drug resistance in non-small cell lung Cancer (NSCLC): impact of genetic and non-genetic alterations on therapeutic regimen and responsiveness. Pharm Therapeutics.

[3] Dostert C, Pétrilli V, Van Bruggen R, Steele C, Mossman BT, Tschopp J (2008). Innate immune activation through Nalp3 inflammasome sensing of asbestos and silica. Science..

[4] Colarusso C, Terlizzi M, Molino A, Imitazione P, Somma P, Rega R, Saccomanno A, Aquino RP, Pinto A, Sorrentino R (2019). AIM2 Inflammasome activation leads to IL-1α and TGF-β release from exacerbated chronic obstructive pulmonary disease-derived peripheral blood mononuclear cells. Front Pharmacol.

[5] Engels EA (2008). Inflammation in the development of lung cancer: epidemiological evidence. Expert Rev Anticancer Ther.

[6] Dinarello CA, van der Meer JW (2013). Treating inflammation by blocking interleukin-1 in humans. Semin Immunol.

[7] Landvik NE, Hart K, Haugen A, Zienolddiny S (2012). Functional analysis of a lung cancer risk haplotype in the IL1B gene regulatory region. J Hum Genet.

[8] Terlizzi M, Casolaro V, Pinto A, Sorrentino R (2014). Inflammasome: cancer friend’s or foe?. Pharm Therapeutics.

[9] Viganò E, Mortellaro A (2013). Caspase-11: the driving factor for noncanonical inflammasomes. Eur J Immunol.

[10] Kayagaki N, Warming S, Lamkanfi M, Vande Walle L, Louie S, Dong J, Newton K, Qu Y, Liu J, Heldens S, Zhang J, Lee WP, Roose-Girma M, Dixit VM (2011). Non-canonical inflammasome activation targets caspase-11. Nature..

[11] Terlizzi M, Colarusso C, Popolo A, Pinto A, Sorrentino R (2016). IL-1α and IL-1β-producing macrophages populate lung tumor lesions in mice. Oncotarget..

[12] Terlizzi M, Colarusso C, De Rosa I, De Rosa N, Somma P, Curcio C, Sanduzzi AZ, Micheli P, Molino A, Saccomanno A, Salvi R, Aquino RP, Pinto A, Sorrentino R (2018). Circulating and tumor-associated caspase-4: a novel diagnostic and prognostic biomarker for non-small cell lung cancer. Oncotarget..

[13] Terlizzi M, Di Crescenzo VG, Perillo G, Galderisi A, Pinto A, Sorrentino R (2015). Pharmacological inhibition of Caspase-8 limits lung tumor outgrowth. Br J Pharmacol.

[14] Sorrentino R, Morello S, Chen S, Bonavita E, Pinto A (2010). The activation of liver X receptors inhibits toll-like receptor-9-induced foam cell formation. J Cell Physiol.

[15] van de Veerdonk FL, Netea MG, Dinarello CA, Joosten LA (2011). Inflammasome activation and IL-1β and IL-18 processing during infection. Trends Immunol.

[16] Kim JW, Koh Y, Kim DW, Ahn YO, Kim TM, Han SW, Oh DY, Lee SH, Im SA, Kim TY, Heo DS, Bang YJ (2013). Clinical implications of VEGF, TGF-β1, and IL-1β in patients with advanced non-small cell lung Cancer. Cancer Res Treat.

[17] Wu C, Xu B, Zhou Y, Ji M, Zhang D, Jiang J, Wu C (2016). Correlation between serum IL-1β and miR-144-3p as well as their prognostic values in LUAD and LUSC patients. Oncotarget..

[18] Terlizzi M, Molino A, Colarusso C, Donovan C, Imitazione P, Somma P, Aquino RP, Hansbro PM, Pinto A, Sorrentino R (2018). Activation of the Absent in Melanoma 2 Inflammasome in Peripheral Blood Mononuclear Cells From Idiopathic Pulmonary Fibrosis Patients Leads to the Release of Pro-Fibrotic Mediators. Front Immunol.

[19] Chanvorachote P, Sriratanasak N, Nonpanya N (2020). C-myc contributes to malignancy of lung Cancer: a potential anticancer drug target. Anticancer Res.

[20] Kortlever RM, Sodir NM, Wilson CH, Burkhart DL, Pellegrinet L, Brown Swigart L, Littlewood TD, Evan GI (2017). Myc Cooperates with Ras by Programming Inflammation and Immune Suppression. Cell.

[21] Kayagaki N, Wong MT, Stowe IB, Ramani SR, Gonzalez LC, Akashi-Takamura S, Miyake K, Zhang J, Lee WP, Muszyński A, Forsberg LS, Carlson RW, Dixit VM (2013). Noncanonical inflammasome activation by intracellular LPS independent of TLR4. Science..

[22] Poltorak A, He X, Smirnova I, Liu MY, Van Huffel C, Du X, Birdwell D, Alejos E, Silva M, Galanos C, Freudenberg M, Ricciardi-Castagnoli P, Layton B, Beutler B (1998). Defective LPS signaling in C3H/HeJ and C57BL/10ScCr mice: mutations in Tlr4 gene. Science..

[23] Hou L, Sasaki H, Stashenko P (2000). Toll-like receptor 4-deficient mice have reduced bone destruction following mixed anaerobic infection. Infect Immun.

[24] Riccardo F, Arigoni M, Buson G, Zago E, Iezzi M, Longo D, Carrara M, Fiore A, Nuzzo S, Bicciato S, Nanni P, Landuzzi L, Cavallo F, Calogero R, Quaglino E (2014). Characterization of a genetic mouse model of lung cancer: a promise to identify non-small cell lung Cancer therapeutic targets and biomarkers. BMC Genomics.

[25] Sorrentino R, Terlizzi M, Di Crescenzo VG, Popolo A, Pecoraro M, Perillo G, Galderisi A, Pinto A (2015). Human lung Cancer-derived Immunesuppressive Plasmacytoid dendritic cells release IL-1α in an AIM2 Inflammasome-dependent manner. Am J Pathol.

[26] Cheng KT, Xiong S, Ye Z, Hong Z, Di A, Tsang KM, Gao X, An S, Mittal M, Vogel SM, Miao EA, Rehman J, Malik AB (2017). Caspase-11-mediated endothelial pyroptosis underlies endotoxemia-induced lung injury. J Clin Invest.

[27] Maes M, Vanhaecke T, Cogliati B, Yanguas SC, Willebrords J, Rogiers V, Vinken M (2015). Measurement of Apoptotic and Necrotic Cell Death in Primary Hepatocyte Cultures. Methods Mol Biol.

[28] Issa R, Sorrentino R, Sukkar MB, Sriskanden S, Fan Chung K, Mitchel JA (2008). Differential regulation of CCL-11/eotaxin-1 and CXCL-8/IL-8 by gram-positive and gram-negative bacteria in human airway smooth muscle cells. Respir Res.

[29] Forte G, Rega A, Morello S, Luciano A, Arra C, Pinto A, Sorrentino R (2012). Polyinosinic-polycytidylic acid limits tumor outgrowth in a mouse model of metastatic lung cancer. J Immunol.

[30] Méry B, Guy JB, Vallard A, Espenel S, Ardail D, Rodriguez-Lafrasse C, Rancoule C, Magné N (2017). In vitro cell death determination for drug discovery: a landscape review of real issues. J Cell Death.

[31] Terlizzi M, Molino A, Colarusso C, Somma P (2020). De rosa I, Troisi J, Scala G, Salvi R, Pinto A and Sorrentino R. Altered lung tissue lipidomic profile in caspase-4 positive non-small cell lung cancer (NSCLC) patients. Oncotarget..

[32] Koukourakis MI, Giatromanolaki A, Sivridis E, Gatter KC, Harris AL (2005). Tumor and Angiogenesis Research Group. Pyruvate dehydrogenase and pyruvate dehydrogenase kinase expression in non small cell lung cancer and tumor-associated stroma. Neoplasia..

[33] Marquez J, Flores J, Kim AH, Nyamaa B, Nguyen ATT, Park N, Han J. Rescue of TCA Cycle Dysfunction for Cancer Therapy. J Clin Med. 2019;8(12). 10.3390/jcm8122161 PMC6947145 31817761

[34] Trinidad AG, Whalley N, Rowlinson R, Delpuech O, Dudley P, Rooney C, Critchlow SE (2017). Pyruvate dehydrogenase kinase 4 exhibits a novel role in the activation of mutant KRAS, regulating cell growth in lung and colorectal tumour cells. Oncogene..

[35] Patra KC, Wang Q, Bhaskar PT, Miller L, Wang Z, Wheaton W, Chandel N, Laakso M, Muller WJ, Allen EL, Jha AK, Smolen GA, Clasquin MF, Robey B, Hay N (2013). Hexokinase 2 is required for tumor initiation and maintenance and its systemic deletion is therapeutic in mouse models of cancer. Cancer Cell.

[36] Camarda R, Williams J, Goga A (2017). In vivo Reprogramming of Cancer Metabolism by MYC. Front Cell Dev Biol.

[37] Papoff G, Presutti D, Lalli C, Bolasco G, Santini S, Manelfi C, Fustaino V, Alemà S, Ruberti G (2018). CASP4 gene silencing in epithelial cancer cells leads to impairment of cell migration, cell-matrix adhesion and tissue invasion. Sci Rep.

