# Supplementary figures and images for: Identification of a novel subpopulation of Caspase-4 positive non-small cell lung Cancer patients
Source: J Exp Clin Cancer Res. 2020 Nov 13;39:242. doi: 10.1186/s13046-020-01754-0 (PMC7664047; doi:10.1186/s13046-020-01754-0)

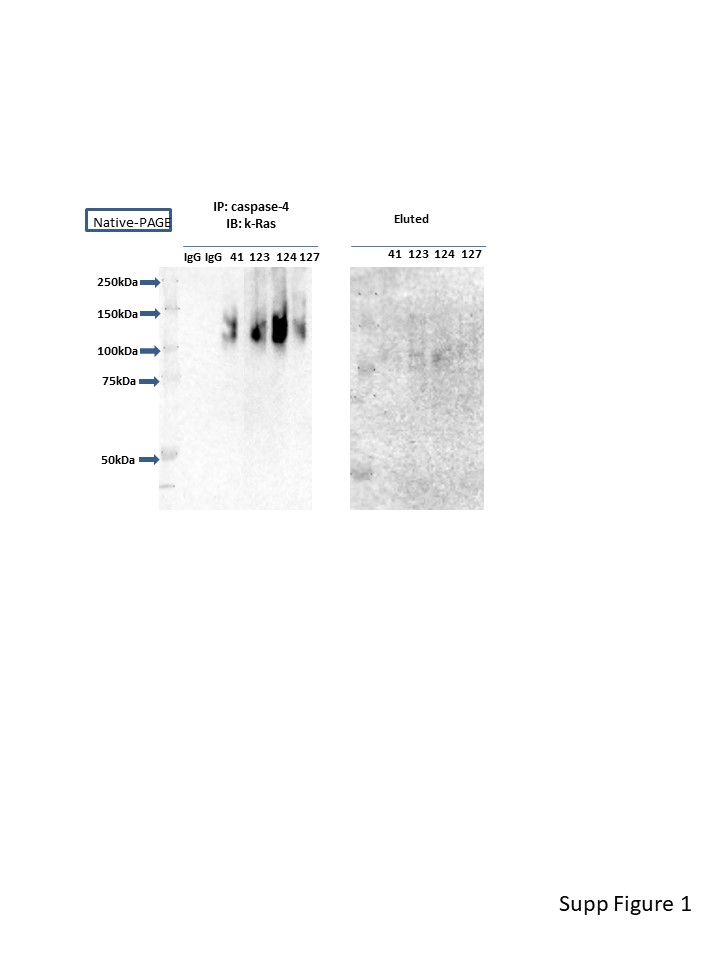

Supplement: Supplementary file 1 — Additional file 1: Fig. S1. Human caspase-4 co-immunoprecipitated with k-RAS in human tumor samples. Human digested lung tumor samples were analyzed by means of Native-PAGE. The isotype control of the antibody against caspase-4 did not show any aspecific band. Similarly eluted samples did not show any positive band, implying that caspase-4 and K-RAS are bound in human lung tumor samples obtained by NSCLC patients. Experiments were performed three times. [file 13046_2020_1754_MOESM1_ESM.jpg]
